# Vigilance to misleading information is required to avoid delayed diagnosis: Case series of acral melanomas

**DOI:** 10.1016/j.amsu.2021.102270

**Published:** 2021-04-21

**Authors:** Sumadi Lukman Anwar, Ery Kus Dwianingsih, Tania Maharani Chandra, Arini Rizky Wijayanti, Haryo Widhanto, Adryan Kalya Ndraha Khairindra, Herjuna Hardiyanto, Suwardjo Suwardjo

**Affiliations:** a Division of Surgical Oncology - Department of Surgery, Dr Sardjito Hospital / Faculty of Medicine, Public Health, and Nursing, Universitas Gadjah Mada, Yogyakarta, 55281, Indonesia; b Department of Anatomical Pathology, Dr Sardjito Hospital / Faculty of Medicine, Public Health, and Nursing, Universitas Gadjah Mada, Yogyakarta, 55281, Indonesia

**Keywords:** Melanoma, Acral, History taking, Misleading, Delayed diagnosis

## Abstract

**Introduction:**

Melanoma is considered a rare cancer among Asians with a wide range of mucocutaneous manifestations. Failure to recognize a lesion as melanoma at first presentation might delay surgery aimed at complete resection. Acral melanoma has been related with the highest rate of misdiagnosis (~30%) causing further delayed diagnosis. Reliability of patient’ history taking in melanoma has not yet been systematically reported.

**Presented cases:**

Two patients visited our oncology clinic with pigmented lesions in their soles. A 66-year-old man disclosed it appeared since a year ago after accidently hitting a stone while farming. Physical examination showed a black-brown irregular 100 × 80 mm lesion covering the distal third of the right sole with ulceration in the central lesion. The second patient was a geriatric woman with a black-purple 25 × 27 mm lesion with slight protrusion and ulceration in the central, irregular border, and partial hyperkeratosis. She explained the lesion emerged two years ago after she accidently stepped on a nail. Both patients were then diagnosed with acral melanomas and were treated with wide-excision, closure with skin grafting, and inguinal dissection.

**Discussion:**

Both patients reported history of traumas in lesions later confirmed as acral melanomas. Although history taking can provide up to 80% of the information for accurate diagnosis, in ambivalent cases, careful anamnesis, clinical examination, and biopsy are required to confirm diagnosis of acral melanoma. Early disease identification to establish definitive diagnosis of cancer is generally associated with better clinical outcomes. In suspected cases, vigilance toward misleading information in history taking is required.

## Highlights

• Acral melanoma is associated with higher rates of misdiagnosis than other melanoma subtypes.
• Elderly patients often have chronic podiatric lesions associated with history of trauma.
• Caution concerning misleading information during history taking is required to avoid delayed diagnosis of melanoma.
• Biopsy is warranted for all undetermined skin lesions including unresolved wounds after standard treatment.

## 1 Introduction

Cutaneous melanoma is the most aggressive form of skin cancers with annual incidence of 232,100 cases worldwide [1]. Although melanoma accounts for only 1% of all skin cancers, it causes the majority of skin cancer-related mortality [2]. Melanoma is exceptionally rare among Asians [3], which contributes to the relatively low public awareness about the disease [4]. Disease presentation of melanoma can differ among different ethnicities [2]. Clinical manifestations of cutaneous melanoma in people of color, specifically acral lentiginous melanoma (ALM), are overrepresented and often appear in hard-to-find places including toenails, fingernails, soles, or palms [2,3].

The diagnosis of acral melanoma is often delayed since the predilection area of this subtype of melanoma is not regularly examined by the patients nor general practitioners [4]. The lesions in many instances present as a pigment deficient entity causing less suspicion than melanoma [3]. Acral melanoma lesions are also often mistaken as another skin pathology, such as infection and vascular lesions. The range of skin lesions in the feet including nevus, subungual hemorrhage, verruca, granuloma, onychomycosis, and diabetic ulcers should include ALM as one of the differential diagnoses [4,5]. Among melanoma subtypes, ALM is associated with a higher misdiagnosis rate of 25–36% [6], causing further delay in diagnosis and treatment initiation. Early disease identification to establish definitive diagnosis of cancer is generally associated with better clinical outcome. In cutaneous melanoma, prognosis is mainly determined by Breslow's tumor thickness of the primary lesion and locoregional spread. Melanoma cases among Asians are predominantly diagnosed in advanced stages [3,7], causing difficulty in achieving complete resection.

Failure to recognize a lesion as melanoma at the patient's first visit will contribute to delayed diagnosis and initial treatment, causing increased risk for potential distant organ metastases and premature mortality due to the cancer [6]. Associated factors for delayed diagnosis of melanoma derive from both patients and the health system [2]. Higher level of awareness about melanoma has been reported to elude delays in establishing diagnosis and treatment initiation. Misdiagnosis due to improper confidence concerning collecting information and physical examination might also exacerbate the delay [6,8]. Thorough visual examination particularly direct inspection and following an appropriate algorithm in the clinical pathway have been associated with an increased sensitivity to accurately diagnosis melanoma [8]. In addition to physical examination, history taking also plays a vital role in establishing accurate cancer diagnosis. Research systematically investigating the role of accuracy in anamnesis in preventing misdiagnosis or delayed diagnosis of ALM is lacking. Considering the minor association between trauma and cutaneous melanoma [9] and the expected frequency of trauma to the extremities [6], we present two geriatric cases to underscore the importance for general practitioners in the primary health care and specialists in oncology clinics to maintain vigilance toward misleading information during history taking in patients with suspected skin lesions. This case series was presented following the **P**referred **R**eporting **O**f **C**as**E S**eries in **S**urgery (PROCESS) guidelines [10].

## 2 Presented case

**Fig. 1:**
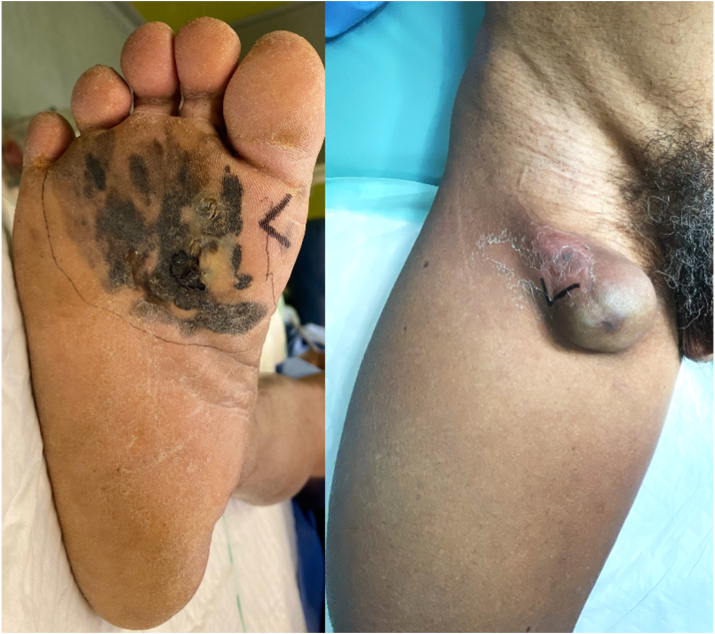
A 66-year-old man presented in the polyclinic with a black-brown irregular 100 × 80 mm skin lesion fulfilling the distal third of the right sole with ulceration in the central lesion. Nodular-shaped, purple to black lump of 3.5 cm in diameter was also observed in the right inguinal region. (For interpretation of the references to color in this figure legend, the reader is referred to the Web version of this article.)

**Fig. 2:**
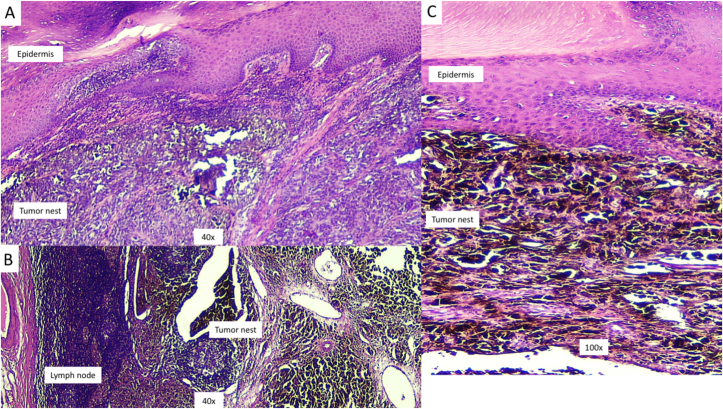
Histopathology of the skin lesion demonstrates accumulation of round, oval, epithelioid, to pleomorphic tumor cells with of intra- and extracellular melanocytic pigments that infiltrate the epidermis and surrounding soft tissues (A,C). The histological features meet the growth pattern of acral lentiginous melanoma (ALM). In the panel B, infiltration of cancer cells is observed in the lymph nodes with prominent extra-nodal extension.

A 66-year-old man came to the oncology clinic with a pigmented lesion on his right sole. The patient had no medical comorbidity or any history of cancer. According to the patient, the lesion became apparent after accidently hitting a stone while farming about one year before presentation at the clinic. Upon inspection of the right foot, a black-brown irregular 100 × 80 mm lesion was found covering the distal third of the right sole (Fig. 1). In addition, a nodular-shaped, purple-black lump of 3.5 cm in diameter was also observed in the right inguinal region that was confirmed using sonography. The patient underwent wide excision to remove the lesion, followed by skin-graft wound closure, and inguinal lymph node dissection. Histopathology examination revealed round, oval, epithelioid to pleomorphic tumor cells with intra- and extracellular melanocytic pigment that infiltrated the epidermis and surrounding soft tissues (Fig. 2). Breslow thickness was 12 mm, and the five inguinal lymph nodes were infiltrated with the tumor cells and an evidence of extra-nodal extension (pT4aN3M0).

**Fig. 3:**
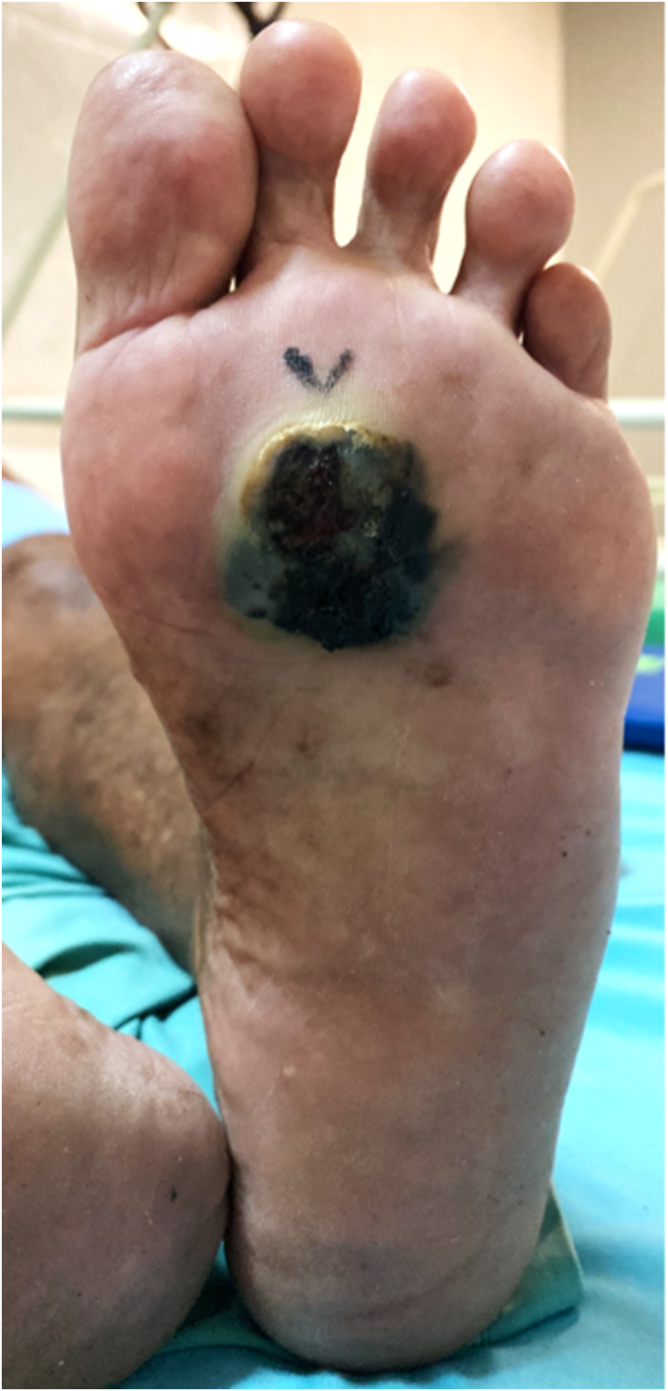
Clinical features of skin lesion in the left sole of a 76-year-old woman who stated that the lesion appeared after she accidently stepped on a nail one year ago. Physical examination showed a 25 × 27 mm purplish-black lesion with slight protrusion and central ulceration. The lesion has irregular border and partial hyperkeratosis.

**Fig. 4:**
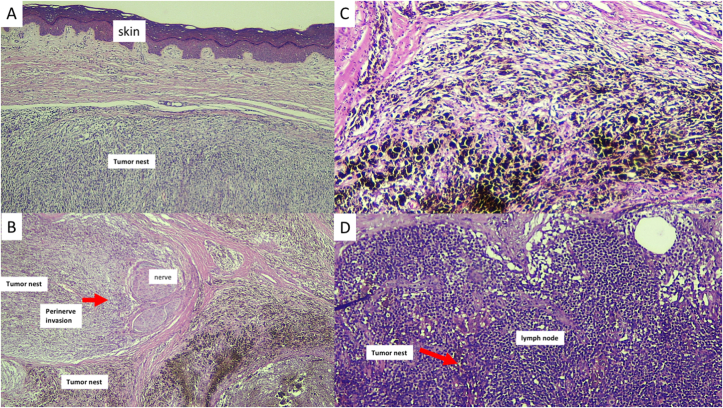
Histopathology of the lesion shows dense proliferation of atypical melanocytes that are specifically arranged in nodular and solid patterns. The tumor cells are sharply circumscribed without Pagetoid migration and lateral extension to the epidermis (A and C). Perineural invasion is observed in the Panel B, and tumor cell infiltration is shown in the Panel D.

One week later, a 76-year-old woman presented to the oncology clinic with a pigmented lesion on her left sole. She had no medical comorbidity nor history of cancer. The patient said that the lesion appeared two years ago after she accidently stepped on a nail. Careful inspection showed a 25 × 27 mm purplish-black lesion with slight protrusion and central ulceration. The lesion had an irregular border and partial hyperkeratosis (Fig. 3). Wide excision, skin-graft wound closure, and inguinal lymph node dissection were done to treat the patient. Histopathology examination showed dense melanocytic proliferation with significant variable cytologic atypia and was arranged in nodular and solid patterns infiltrating to the surrounding soft tissue (Fig. 4). The maximum tumor thickness was 13.3 mm, and the three inguinal lymph nodes were positive for tumor cells (pT4bN2M0).

Both patients received adjuvant radiotherapy and were monitored in the hospital setting. They were able to return to their daily activities six months after surgery.

## 3 Discussion

Acral melanoma has been frequently associated with poorer outcome than other melanoma subtypes [11]. The genetic landscapes of acral melanoma are strikingly different from other sun damage-associated melanoma by showing lower mutational loads, more chromosomal structural changes, and lower levels of cytosine to thymine mutations [12]. Although mutation signatures contribute to the clinical course and treatment response, relatively poorer outcome of acral melanoma is associated with the delayed presentation at diagnosis [11]. The mainstay of treatment in early stages of melanoma is surgical excision with sufficient margin. To be able to achieve complete resection and prevent functional compromise [13,14], early detection is required to identify the disease before it spreads to the regional lymph nodes and distant organs.

Cutaneous melanoma is regarded as a rare cancer among Asians [3,7]. The most common predilection site for cutaneous melanoma in Asians is in the sole [3,7], an area that is often neglected during general check-up. Low community awareness and thicker Breslow at diagnosis are associated with delayed diagnosis and poor outcome of acral melanoma [4,15]. Sondermann et al. reported the relatively high rate of misdiagnosis (~30%) in ALM [13]. Other reports also showed around 25–33% initial incorrect diagnosis of acral melanoma [16,17], which has also been associated with adverse outcome and prognosis [5,13]. Acral lesions with ulceration are also often misinterpreted as infection origin. Around a third of acral melanoma cases were first evaluated as an infection [6]. Recognizing melanoma can also be challenging due to the variability of its clinical presentations (Figs. 1 and 3). Some differential diagnoses of melanotic acral lesions are hemangioma, hematoma, nevus, warts, fungal infection, diabetic foot ulcer, pyogenic granuloma, and angiosarcoma. Low cancer awareness and the variability of clinical presentations potentially can contribute to misdiagnosis or delayed diagnosis of acral melanoma which then leads to increased thickness of the tumor and worse prognosis.

Areas such as soles, palms, and nails are prone to repeated injuries [6]. Accordingly, skin lesions are also frequently misinterpreted as chronic wounds from posttraumatic injury, diabetic foot lesions, and peripheral occlusive arterial diseases because of the high prevalence of these diseases among elderly patients [6]. Careful history taking and detailed physical examination are required to rule out the potential of missed or misinformation during the first meeting with patients [8]. Because melanoma presents with its marked variety and visibility that might resemble benign skin lesions, accurate anamnesis is very important in establishing diagnosis and preventing worse prognosis. History taking can provide as much as 80% of the information needed for diagnosis as in other medical fields. A structured approach is usually needed to obtain all necessary information from the patients [6,8]. During the first visit, demographic information including age, sex, ethnic, occupation, and residence are important points to identify some associated risk factors, while focusing on the primary lesion, duration of onset, provoking and relieving exposures, exacerbation, and evolving conditions. Detailed locations and associated symptoms including itch, tenderness, inflammation, discharge, bleeding, and accompanying systemic symptoms are very important during anamnesis. In addition, response to previous treatment, past medical history, allergy, family history of cancer, smoking, sun exposure and other associated risk factors need to be documented [6,8].

History taking can be substantially refined by giving focused questions followed with attentive listening [8]. However, the patient's affirmation of past injury often tends to be off target in the recognition of podiatric melanoma. Our cases showed confounding anamnesis as patients had difficulty in remembering and reporting the history of trauma or hematoma in the suspected lesions. Albreski et al. also showed that more than 35% of misdiagnosis of lesions in lower extremities were due to trauma [6]. Particularly in elderly patients, history of trauma in chronic lesions should be explored carefully. Another report showed that failure to accurately diagnosis melanoma had occurred for a chronic wound that was reported as trauma in a patient with family history of cancer [18]. Personal or family history of cancer might also substantiate for further need of a biopsy. In addition to history taking, accuracy of melanoma diagnosis also depends on the direct visual and image-based visual inspection. Direct visual observation followed by further examination according to the clinical pathway results in better sensitivity to definitively diagnose melanoma [8]. With the wide variations possible in visual inspection, further study is required to improve accuracy and application of the clinical checklists used for physical examination.

Under the current universal health coverage in Indonesia, anamnesis and clinical examination of suspected skin lesions are usually performed by general practitioners who will decide about referral for further examination by dermatologists or oncologists [19]. The ability to recognize melanoma might vary according to their experience and training. There are also particularly different roles between general practitioners and specialists which might complicate the effectivity of the health referral system. General practitioners must urgently refer patients with suspected lesions but are also expected to limit unnecessary referral. Meanwhile, specialists must identify high risk lesions for urgent excision or biopsy, provide further adjuvant treatment, and surveillance. In the presence of misleading information during history taking, pattern recognition and clinical reasoning could be diminished [8]. In potentially aggressive cancer including cutaneous melanoma, misinterpreting history taking might cause delayed diagnosis and reduce the opportunity for complete resection [13]. In our series, since the two cases were diagnosed during the COVID-19 pandemic, the inability to recognize them as melanoma might also cause significant impacts because most of the non-essential elective surgeries were frequently postponed during 2020 and in the post-peak pandemic period [20,21].

In all suspected and undetermined lesions, a biopsy should always be considered. Among acral melanomas that are also commonly misdiagnosed are the rare cases of ungual melanoma [22]. However, melanonychia striata is frequently found in Asian and African elderly patients. Pigmentation in the nail or nail-fold should be evaluated, and a biopsy done in the presence of evolving or deteriorating conditions [6,22]. Ungual or subungual hemorrhages that do not resolve after podiatric treatment or shoe modification should warrant further examination or biopsy. It is estimated that re-epithelialization in a wide podiatric wound will take around eight weeks. Topical medication or systemic treatment should be thoroughly evaluated in lesions without immediate improvement. Histologic evaluation is required to confirm all clinically suspected cases including the featureless lesions and those without substantial healing under adequate treatment [23]. Definitive diagnosis of ALM can be clearly confirmed histologically by accumulation of melanocytes overlying the nest within the epidermis and spindle-shaped melanocytes in the dermis while lacking epidermal atrophy and melanocytes in the upper layer of the epidermis [24] as shown in Fig. 2. Although rarely reported, the nodular type of acral melanoma showing melanocytic proliferation arranged in nodular pattern and sharply circumscribed without lateral extension (Fig. 4) can be found [24].

Our report emphasizes the importance for vigilance toward the existence of any misinformation during history taking in patient with suspected skin lesions. Further study is required to comprehensively analyze the impacts of public awareness and knowledge about skin melanoma as well as the presence of misleading information during history taking with delayed diagnosis, health care delivery, and the outcome. Therefore, early detection is very important to find and diagnose melanoma in early stages to facilitate complete resection and to improve the patient's survival.

## 4 Conclusion

Our two patients in this report both described a history of trauma on the location of the lesions, which could mislead the physician into making the simple diagnosis of superficial injury. Because delayed diagnosis can lead to advanced stages at first primary treatment and poor prognosis, all skin lesions that do not respond to standard wound care, patient-reported trauma, and nonhealing lesions should increase the suspicion of melanoma and warrant further examination of biopsy.

## Declaration of competing interest

We declare that no potential conflict of interest exists.

## Abbreviations

ALM: acral lentiginous melanoma
AJCC: The American Joint Committee on Cancer
NM: nodular melanoma
PROCESS: Preferred Reporting Of CasE Series in Surgery
SSM: superficial spreading melanoma

## Registration of research studies

The study has been registered in the Research Registry with identification number researchregistry6192. Please find in the https://www.researchregistry.com/browse-the-registry#home/registrationdetails/5f9de98dc4597c0015c1a5f8/.

## Availability of data and materials

The clinical and imaging data supporting the analysis and findings of this study will be available from the corresponding author upon reasonable request.
